# Disorders of acid-base balance promote rumen lipopolysaccharide biosynthesis in dairy cows by modulating the microbiome

**DOI:** 10.3389/fmicb.2024.1492476

**Published:** 2024-11-14

**Authors:** Guobin Hou, Jingtao You, Yimin Zhuang, Duo Gao, Yiming Xu, Wen Jiang, Sumin Li, Xinjie Zhao, Tianyu Chen, Siyuan Zhang, Shuai Liu, Wei Wang, Shengli Li, Zhijun Cao

**Affiliations:** ^1^State Key Laboratory of Animal Nutrition and Feeding, International Calf and Heifer Organization, College of Animal Science and Technology, China Agricultural University, Beijing, China; ^2^College of Animal Science, Xinjiang Agricultural University, Urumqi, China

**Keywords:** subacute ruminal acidosis, rumen pH, lipopolysaccharide, microbiome, inflammation

## Abstract

**Introduction:**

Disorders of acid-base balance in the rumen of dairy cows have a significant impact on their health and performance. However, the effect of transient differences in pH on susceptibility to subacute ruminal acidosis (SARA) and lipopolysaccharide (LPS) biosynthesis in dairy cows remains unclear.

**Methods:**

In this study, milk, serum, and rumen fluid samples from 40 Holstein dairy cows (on d 56 postpartum) with different rumen pH (2–4 h after morning feeding) were explored to investigate the difference of susceptibility to SARA and the correlation between microbiome, LPS and inflammation. These cows were categorized into low pH (LPH, pH ≤ 6.0, *n* = 20) and high pH (HPH, pH ≥ 6.5, *n* = 20) groups.

**Results:**

The results showed that LPH group increased the concentrations of total volatile fatty acids, acetate, propionate, butyrate and valerate. However, milk yield and milk compositions were unaffected. Compared to the HPH group, the LPH group increased the concentrations of serum BHBA, NEFA, LPS, HIS, IL-2, IL-6, TNF-α, and MDA, and decreased the concentrations of serum IgA, IgM, IgG, SOD, T-AOC, and mTOR. In addition, the LPH group decreased the copies of *Ruminococcus flavefaciens* and increased the copies of *Fibrobacter succinogenes*. Microbial community analysis isupplendicated a significant difference in bacterial composition between the two groups. At the phylum level, Bacteroidota and Firmicutes were enriched in the LPH and HPH groups, respectively. At the genus level, the dominant bacteria in the LPH group were *Prevotella*. Additionally, the LPH group increased the proportions of Gram-negative phenotypes, potentially pathogenic phenotypes and LPS biosynthesis. The close correlation between two key enzymes for LPS synthesis *LpxL* and *LpxM* with rumen pH, inflammatory markers, and microorganisms indicates that low pH may increase the risk of inflammation by facilitating the lysis of Gram-negative bacteria and the release of penta-acylated LPS. Penta-acylated and hexa-acylated LPS may be mainly derived from *Prevotella* and *Succinivibrionaceae_UCG-001*, respectively.

**Discussion:**

Overall, these results support the notion that transient low pH could reflect the risk of cows suffering from SARA and associated inflammation and is strongly associated with penta-acylated LPS. Our findings provide new insights into ruminant health improvement and disease prevention strategies.

## 1 Introduction

Subacute ruminal acidosis (SARA) is a common metabolic disease in high-yielding dairy cows during the early and middle stages of lactation (Elmhadi et al., 2022; Plaizier et al., 2022). During these periods, cows typically meet their lactation requirements by consuming grain-rich diets. The volatile fatty acids (VFA) produced by fermentable carbohydrates in grains in the presence of starch-degrading bacteria can be utilized as a significant source of energy for dairy cows. However, when the rate of absorption of VFA across the rumen epithelium and small intestine does not match the rate of its production, VFA accumulates in large quantities and causes disturbances in rumen acid-base balance (Zebeli et al., 2008). Impaired rumen epithelial cell function and an imbalance in the ratio of fiber-degrading to starch-degrading bacteria are important features following a decrease in rumen pH and affect feed intake and dry matter catabolism in dairy cows (Bevans et al., 2005; Plaizier et al., 2016). In addition, low rumen pH may also increase the risk of ruminitis, mastitis, laminitis, and endometritis in dairy cows, which is closely related to the release of lipopolysaccharides (LPS) into the peripheral circulation after lysis of gram-negative bacteria (Zhao et al., 2018; Fu et al., 2022; Zeng et al., 2023). These symptoms can be reflected by increased LPS in rumen fluid, blood and milk (Hu et al., 2022; Zhang H. et al., 2023). Indeed, the capacity of LPS with different structures to induce an inflammatory response varies considerably due to differences in the degree of lipid A acylation of LPS (Brix et al., 2015; Dovala et al., 2016). Kdo2-lipid IVA lauroyltransferase/acyltransferase (*LpxL*) and lauroyl-Kdo2-lipid IVA myristoyltransferase (*LpxM*) are two key enzymes in lipid A synthesis (Brix et al., 2015). Therefore, SARA and related inflammation in dairy cows are significantly influenced by changes in the rumen microbiota and LPS.

Currently, the prevalence of SARA has been significantly improved through improved feeding management and the use of nutritional additives, such as sodium bicarbonate, plant extracts and *Saccharomyces cerevisiae* fermentation product, and gallic acid (Li et al., 2016; Plaizier et al., 2022; Rivera-Chacon et al., 2022; Zhao et al., 2024). Nevertheless, despite the uniformity of feeding management, a subset of cows exhibited heightened susceptibility to SARA, manifesting as decreased rumen pH, elevated total VFA and an imbalance in the ratio of starch-degrading bacteria to fiber-degrading bacteria, which are characteristic of SARA (Zhang et al., 2022; Zhang Z. A. et al., 2023). Although the ruminal pH varies in different parts and throughout the day, the lowest pH is typically observed 2–4 h after morning feeding (Li et al., 2009; He et al., 2022; Ma et al., 2022). During this period, a lower rumen pH may increase the risk of SARA, while a higher pH may be associated with lower SARA risk. It is therefore imperative to gain an understanding of the microbial status and host metabolic profiles of SARA-susceptible dairy cows, as this will facilitate further enhancements to cow health and maximize their productive performance.

In this study, we characterized the level of susceptibility of cows to SARA based on rumen fluid pH 2–4 h after morning feeding. The susceptibility of dairy cows to SARA at different pH values was explored by analyzing milk composition, serum indicators, rumen fermentation parameters and microorganisms. Phenotypic and functional analyses of the 16S rRNA gene sequencing results revealed further differences in rumen LPS biosynthesis and its correlation with inflammation at different pH levels.

## 2 Materials and methods

### 2.1 Ethics statement

This study was conducted at the Gansu State Farms Tianmu Dairy Co., Ltd. (Jinchang, Gansu Province, China) from October to November 2022. The experiment and animal procedures were done according to the Guidelines for Care and Use of Laboratory Animals of China Agricultural University (Beijing, China) and approved by the Animal Ethics Committee of China Agricultural University (Approval No. AW10803202-3-2).

### 2.2 Animal experimental design and diet

One hundred healthy early lactation Holstein dairy cows (Parity = 2.73 ± 0.73, BCS = 3.04 ± 0.25 (d 21), Mean ± SD) were selected, and were housed in a well-ventilated barn with lying beds and sand bedding. Throughout the entire experiment (from d 21 to 56 days postpartum), all cows were supplied the same total mixed ration (TMR) three times a day at 07:00, 14:00, and 21:00 and had free access to clean water. The cows were milked three times daily before feeding using rotary milking system. Cows are at their lowest rumen pH 2–4 h after morning feeding. During this period, susceptibility to SARA may be higher at rumen pH below 6.0 and lower at pH above 6.5. The cows were divided into two groups according to pH value of rumen fluid 2–4 h after morning feeding on d 56: low-pH group (LPH, pH ≤ 6.0, *n* = 20) and high-pH group (HPH, pH ≥ 6.5, *n* = 20). Samples with rumen fluid pH between 6.0 and 6.5 were not yet included in this study. A *post hoc* power calculation was performed with G*Power (version 3.1.9.7), the computed power (1 - *β*) = 1.000. The diets are listed in Table 1.

**Table 1:** Ingredients and chemical composition of the diets.

Item	Diet
Ingredient, % of DM	
Corn grain	6.33
Corn silage	48.96
Alfalfa silage	8.77
Soybean meal	4.61
Whole cottonseed	1.23
Cottonseed meal	1.83
Steam-flaked corn	5.31
Molasses	2.65
Beet pulp	1.43
Distillers dried grains with soluble	1.43
Distiller’s grains	13.26
Fat powder	0.98
Fat acids calcium	0.31
Soybean meal pass	1.43
Saleratus	0.21
Methionine	0.04
Premix^a^	1.22
Total	100.00
Nutrient composition, % of DM^b^	
DM, as-fed basis	47.28
CP	17.17
NDF	31.05
ADF	16.59
Starch	29.38
EE	4.64
Ash	7.83

a The premix provided the following per kilogram of diet: 480 mg/kg Cu, 28 mg/kg I, 1,600 mg/kg Mn, 1,800 mg/kg Zn, 13 mg/kg Se, 28 mg/kg Co, 210,000 IU/kg vitamin A, 70,000 IU/kg vitamin D, and 5,600 mg/kg vitamin E.

b DM, dry matter; CP, crude protein; NDF, neutral detergent fiber; ADF, acid detergent fiber; EE, ether extract.

### 2.3 Feed samples

TMR samples were collected weekly and stored at −20°C until chemical composition analysis. The dry matter (DM, Method: 930.15), crude protein (CP, Method: 988.05), ether extract (EE, Method: 920.39), Starch (Method: 996.11) and crude ash (Ash, Method: 924.05) were analyzed based on the Association of Official Analytical Chemists method. The acid detergent fiber (ADF) and neutral detergent fiber (NDF) were analyzed by the ANKOM fiber analyzer (A2000i; American ANKOM, Macedon, NY, United States) (Soest et al., 1991).

### 2.4 Rumen fluid samples

On d 56 relative to parturition, rumen fluid was sampled from each cow through a flexible esophageal tube (2 mm of wall thickness and 18 mm of internal diameter; Anscitech Co., Ltd., Wuhan, Hubei Province, China) at 2–4 h after morning feeding. The ruminal fluid pH was immediately determined with a glass electrode pH meter (pH-100, Lichen, Shanghai, China). Subsequently, the rumen fluid was mixed thoroughly and filtered through 4 layers of cheesecloth. An eight milliliters of rumen fluid were preserved with adding 2 mL of metaphosphoric acid (250 mL/L, volume to volume) and stored at −20°C for determination of volatile fatty acids concentrations and ammonia N concentrations.

The supernatant of rumen fluid sample with sulfuric acid was used to analyze ammonia N concentration, using the assay described by Broderick and Kang (1980). The volatile fatty acids concentrations were measured by an automated gas chromatograph (model 689, Hewlett Packard, Palo Alto, California, United States) equipped with a 0.25-mm internal diameter × 15-m capillary column (Nukol 24106-U, Sulpeco Inc. United States), and the internal standard used was 2-ethylbutyrate. The rest of samples were stored at −80°C for DNA isolation and 16S rRNA gene sequencing and bioinformatics analysis.

### 2.5 Blood samples

All blood samples were collected from 40 cows at d 56 relative to parturition via the tail vein. Blood samples were collected using one 10 mL vacutainer tubes containing no anticoagulant. Samples were centrifuged at 3,500 × *g* at 4°C for 20 min. Serum samples were stored at −20°C until analysis.

The concentration of glucose (GLU) was detected by biochemical method using commercial kits according to the instruction (Beijing Leadman Biochemistry CO., Ltd., Beijing, China). The concentrations of beta-hydroxybutyric acid (BHBA) and non-esterified fatty acid (NEFA) were determined by microplate method using commercial kits (Jiancheng Bioengineering Institute, Nanjing, China). Oxidative stress factors total antioxidant capacity (T-AOC), superoxide dismutase (SOD), glutathione peroxidase (GSH-Px) and malondialdehyde (MDA) were analyzed using microplate, hydroxylamine, colorimetry and thiobarbituric acid methods, respectively (Jiancheng Bioengineering Institute, Nanjing, China). Serum interleukin-1β (IL-1β), interleukin-2 (IL-2), interleukin-6 (IL-6), interleukin-8 (IL-8), interleukin-10 (IL-10), tumor necrosis factor-*α* (TNF-α), interferon-*γ* (IFN-γ), immunoglobulin A (IgA), immunoglobulin M (IgM), immunoglobulin G (IgG), and monocyte chemotactic protein-1 (MCP-1) were analyzed using ELISA kits (Beijing Kangjia Hongyuan Biotechnology Co., Ltd., Beijing, China). Serum lipopolysaccharide (LPS), histamine (HIS), lipopolysaccharide binding protein (LBP), serum amyloid protein A (SAA), phosphatidylinositol 3-kinase (PI3K), protein kinase B (AKT/PKB), mechanistic target of rapamycin (mTOR), and NACHT LRR and PYD domains-containing protein 3 (NLRP3) were analyzed using ELISA kits (Jiancheng Bioengineering Institute, Nanjing, China).

### 2.6 Milk samples

The dairy cows were milked three times a day and the milk yield were recorded at each milking from d 21 to 56 relative to parturition by Dairy Star software. The milk samples (50 mL) were collected on d 56 relative to parturition. Three consecutive milk samples were collected and pooled at a volume ratio based on actual milk weight corresponding to the morning, afternoon, and evening milking, and preserved with potassium dichromate, and stored at 4°C. The concentrations of milk fat, protein, lactose, total solids and somatic cell count were analyzed by the MilkoScan^™^ 7 RM (Foss Analytical, Denmark) and Fossomatic^™^ 7 DC (Foss Analytical, Denmark) within 2 days after sampling.

### 2.7 DNA extraction and quantitative real-time PCR

The bacterial genomic DNA was extracted from rumen fluid samples from LPH (*n* = 20) and HPH (*n* = 20) cows using the Mag Attract Power Soil Pro DNA Kit manual (Qiagen Inc., Germany), following the instruction. The quantification and quality check of the extracted DNA were performed with a Nano-Drop 2000 spectrophotometer (Thermo Fisher Scientific Inc., United States), and then stored at −80°C before further use. The obtained bacterial DNA were used as templates in quantitative real-time PCR (qRT-PCR). The primers that were used in qRT-PCR are listed in Table 2. The qRT-PCR amplification was performed triplicate using Applied Biosystems ^™^ 7300 Real time Fluorescence Quantitative PCR System (Applied Biosystems, United States). The reaction was run in a final volume of 20 μL in 96-well plates; the reaction consisted of 10 μL of 2X ChamQ SYBR Color qPCR Master Mix (Vazyme, Nanjing, China), 0.8 μL of each primer, 0.4 μL of 50 X ROX Reference Dye (ELK Biotechnology, Wuhan, China), 2 μL of bacterial DNA, and 6 μL of ddH_2_O.

**Table 2:** List of primer sequences for quantitative real-time PCR.

	Primer sequences (5′-3′)^a^	Tm (°C)	Product size
*Ruminococcus albus*	F: CCCTAAAAGCAGTCTTAGTTCG R: CCTCCTTGCGGTTAGAAC	56.81	177 bp
		55.05	
*Ruminococcus flavefaciens*	F: TAATACGTAGGGAGCGAGCG	59.13	163 bp
	R: TCACCGCTACACCAGGAATT	59.02	
*Fibrobacter succinogenes*	F: CGCATGGAGGGTTGACTAGA R: GTAGGAGTCTGGGCCGTATC	59.18	155 bp
		59.04	
*Butyrivibrio fibrisolvens*	F: GCCTCAGCGTCAGTAATCG R: GGAGCGTAGGCGGTTTTAC	58.42	188 bp
		58.62	

a F, forward primer; R, reverse primer.

The bacterial DNA of *Ruminococcus flavefaciens*, *Fibrobacter succinogenes*, and *Butyrivibrio fibrisolvens* was amplified the following program: holding at 95°C for 3 min and then 40 cycles of melting at 95°C (5 s), annealing at 58°C (30 s), and extending at 72°C (1 min) consisted of one cycle. The bacterial DNA of *Ruminococcus albus* was amplified the following program: holding at 95°C for 10 min and then 40 cycles of melting at 95°C (15 s), annealing at 58°C (30 s), and extending at 72°C (30 min) consisted of one cycle. Absolute quantification was used for all bacterial DNA assays. The results for counting of each bacterium were expressed as log_10_ copy number of gene copies per mL rumen fluid.

### 2.8 16S rRNA gene sequencing

Bacteria DNA from rumen fluid (LPH & HPH, *n* = 40) was used for 16S rRNA gene sequencing and bioinformatics analysis. The hypervariable region V3–V4 of the bacterial 16S rRNA gene was amplified with primer pairs (338F: 5′-ACTCCTACGGGAGGCAGCAG-3′, 806R: 5′-GGACTACHVGGGTWTCTAAT-3′) using an ABI GeneAmp 9700 PCR thermocycler (ABI, CA, United States) (Liu et al., 2016). The PCR amplicons were sequenced by using an Illumina MiSeq PE300 platform/NovaSeq PE250 platform (Illumina, San Diego, United States) according to the standard protocols by Majorbio Bio-Pharm Technology (Shanghai, China). The raw sequencing reads were deposited into the NCBI Sequence Read Archive database. After demultiplexing, the resulting sequences were quality filtered with Fastp (Chen et al., 2018) and merged with FLASH (Magoč and Salzberg, 2011), according to the overlap relationship between the double-ended reads. Then DADA2’s sequence denoising method was used to process the optimized data and obtain the representative sequence and abundance information of Amplicon Sequence Variants (ASVs). Based on the representative sequence and abundance information of ASVs, a series of statistical or visual analysis such as taxonomic analysis, community diversity analysis, species difference analysis, correlation analysis and functional prediction analysis can be carried out. The data were analyzed on the online platform of Majorbio Cloud Platform (http://www.majorbio.com) (Han et al., 2024).

### 2.9 Statistical analysis

Rumen fermentation parameters, milk composition, cellulolytic bacteria and serum indices were analyzed using independent sample *t*-test in SPSS 26.0 Statistics for Windows (IBM Corp., New York, NY, United States). All data are expressed as the means ± SEM. *p* < 0.05 was considered to indicate a significant difference, *p* < 0.01 an extremely significant difference, and *p* > 0.05 no significant difference.

Based on the ASVs information, the alpha diversity indices including ACE index, Chao index, Shannon index and Simpson index were compared using Wilcoxon rank-sum test with boot (version 1.3.18) and stats package (version 3.3.1) of R (version 3.3.1). Beta diversity was determined to compare the bacterial structure between groups with Bray Curtis dissimilarity and visualized by principal co-ordinates analysis (PCoA) in the R (version 3.3.1). Analysis of similarities (ANOSIM) using the vegan package of R with 9,999 permutations was used to detect the dissimilarities between groups. Community composition analysis completed using Python (version 2.7). The linear discriminant analysis (LDA) effect size (LEfSe) was performed using R (version 3.3.1) to identify the significantly abundant taxa of bacteria among the different groups (LDA score > 3.5). The Kyoto Encyclopedia of Genes and Genomes (KEGG) functional prediction analysis were conducted using PICURST2 functional prediction software (Version 2.2.0-b, http://huttenhower.sph.harvard.edu/galaxy). The Spearman correlation heatmaps between rumen pH, *LpxL*, *LpxM*, serum LPS, inflammatory markers and microorganisms were analyzed and visualized using the pheatmap package (version 1.0.8) and vegan package (version 2.4.3) of R (version 3.3.1) and Python (version 2.7). The thresholds of Spearman correlations were *r* > 0.4 and *p*-value ≤ 0.05. Phenotypic differences between groups were analyzed and visualized using Wilcoxon rank-sum test and BugBase phenotype prediction (https://bugbase.cs.umn.edu/index.html).

## 3 Results

### 3.1 Rumen fermentation parameters and milk composition

Primarily, based on the rumen fluid pH values, the dairy cows were divided into LPH and HPH groups (5.91 ± 0.17 vs. 6.72 ± 0.13, *p* < 0.001). Analysis of rumen fermentation parameters showed that the concentrations of total VFA, acetate, propionate, butyrate and valerate were higher in the rumen of the LPH group (Table 3; *p* < 0.001), while the proportions of isobutyrate (*p* < 0.001) and isovalerate (*p* = 0.032) were lower in the LPH group. Additionally, no significant differences were observed in milk yield, milk fat, milk protein, milk lactose, solid, somatic cell count and fat-to-protein ratio between the LPH and HPH groups (Table 4).

**Table 3:** Rumen fermentation parameters between the LPH and HPH groups.

Item	Groups^a^		SEM	*p*-value
	LPH	HPH		
pH	5.91	6.72	0.07	<0.001
Total VFA (mmol/L)	105.86	78.34	3.47	<0.001
Ammonia-N (mmol/L)	7.89	7.22	0.20	0.087
Acetate (mmol/L)	51.26	39.00	1.62	<0.001
Propionate (mmol/L)	32.69	22.98	1.21	<0.001
Isobutyrate (mmol/L)	1.07	0.99	0.04	0.339
Butyrate (mmol/L)	16.86	12.21	0.60	<0.001
Isovalerate (mmol/L)	1.57	1.36	0.07	0.105
Valerate (mmol/L)	2.40	1.80	0.09	<0.001
Acetate-to-propionate ratio	1.59	1.71	0.04	0.102
Acetate (%)	48.58	49.68	0.38	0.152
Propionate (%)	30.76	29.42	0.41	0.105
Isobutyrate (%)	1.01	1.25	0.04	<0.001
Butyrate (%)	15.90	15.59	0.19	0.426
Isovalerate (%)	1.49	1.74	0.06	0.032
Valerate (%)	2.26	2.32	0.06	0.616

a LPH, low-pH group (pH ≤ 6.0); HPH, high-pH group (pH ≥ 6.5).

**Table 4:** The milk yield and components between the LPH and HPH groups.

Items	Groups^a^		SEM	*p*-value
	LPH	HPH		
Milk yield (kg/d)	47.84	46.72	1.09	0.613
Milk fat (%)	4.01	4.03	0.10	0.950
Milk protein (%)	3.28	3.33	0.04	0.588
Milk lactose (%)	5.28	5.33	0.02	0.218
Solid (%)	14.76	14.86	0.14	0.743
Somatic cell count (×10^3^ cells/mL)	63.20	53.10	12.55	0.751
Fat-to-protein ratio	1.23	1.21	0.03	0.808

a LPH = low-pH group (pH ≤ 6.0), HPH = high-pH group (pH ≥ 6.5).

### 3.2 Serum biochemical, immune, oxidative stress and PI3K signaling pathway related indicators

As shown in Table 5, the concentrations of GLU, SAA, and LBP were not affected significantly by the rumen fluid pH. Compared with HPH group, the concentrations of BHBA (*p* = 0.013), NEFA (*p* < 0.001), LPS (*p* = 0.010), and HIS (*p* = 0.001) were higher in the LPH group, while the concentrations of IgA (*p* < 0.001), IgM (*p* < 0.001), and IgG (*p* = 0.002) were lower in the LPH group. Furthermore, we investigated the existence of notable cytokine discrepancies between the two groups. No significant differences were observed in IL-1β, IL-8, IL-10, IFN-*γ*, and MCP-1 between the LPH and HPH groups, while there was a tendency for IL-1β (*p* = 0.094) to increase in the LPH group. Interestingly, the concentrations of some proinflammatory cytokines were higher in the LPH group, such as IL-2 (*p* = 0.049), IL-6 (*p* = 0.006), and TNF-*α* (*p* = 0.014). Besides, the results of oxidative stress indicators showed no significant difference in the level of GSH-Px between the LPH and HPH groups, but the levels of T-AOC (*p* = 0.042) and SOD (*p* = 0.004) were lower in the LPH group, while the level of MDA (*p* = 0.001) was higher. The results of the assay of indicators related to the PI3K signaling pathway demonstrated that there were no significant differences in PI3K, AKT, and NLRP3 between the LPH and HPH groups. Nevertheless, a reduction in pH resulted in a decline in the serum concentration of mTOR (*p* = 0.005).

**Table 5:** The serum concentrations of biochemical, immune, oxidative stress and PI3K signaling pathway related indicators.

Items	Groups^a^		SEM	*p*-value
	LPH	HPH		
GLU (mmol/L)	3.74	3.85	0.10	0.606
BHBA (mmol/L)	0.47	0.43	0.01	0.013
NEFA (mmol/L)	1.36	1.04	0.05	<0.001
SAA (μg/ml)	194.20	195.75	1.51	0.612
LPS (EU/ml)	0.032	0.027	0.001	0.010
LBP (μg/ml)	152.93	168.50	4.53	0.086
HIS (μg/L)	27.06	23.05	0.63	0.001
IgA (g/L)	0.87	1.10	0.03	<0.001
IgM (g/L)	0.89	0.99	0.01	<0.001
IgG (g/L)	7.49	8.63	0.19	0.002
IL-1β (pg/ml)	28.95	25.73	0.96	0.094
IL-2 (pg/ml)	51.32	47.21	1.06	0.049
IL-6 (pg/ml)	77.23	62.33	2.79	0.006
IL-8 (pg/ml)	15.57	15.00	0.46	0.545
IL-10 (pg/ml)	18.88	19.80	0.83	0.590
TNF-α (pg/ml)	86.88	78.44	1.75	0.014
IFN-γ (pg/ml)	88.17	77.59	3.31	0.111
MCP-1 (pg/ml)	35.40	35.28	1.37	0.964
T-AOC (mmol/L)	0.326	0.342	0.004	0.042
SOD (mU/L)	157.23	162.71	0.99	0.004
GSH-Px (mU/L)	198.20	205.21	4.30	0.422
MDA (μmol/L)	3.15	2.45	0.11	0.001
PI3K (mU/L)	84.29	85.96	0.53	0.120
AKT (μmol/L)	7.87	7.59	0.16	0.390
mTOR (μg/L)	19.64	20.34	0.13	0.005
NLRP3 (μg/L)	2.31	2.25	0.03	0.382

a LPH, low-pH group (pH ≤ 6.0); HPH, high-pH group (pH ≥ 6.5).

### 3.3 The composition of rumen bacteria communities

**Figure 1:**
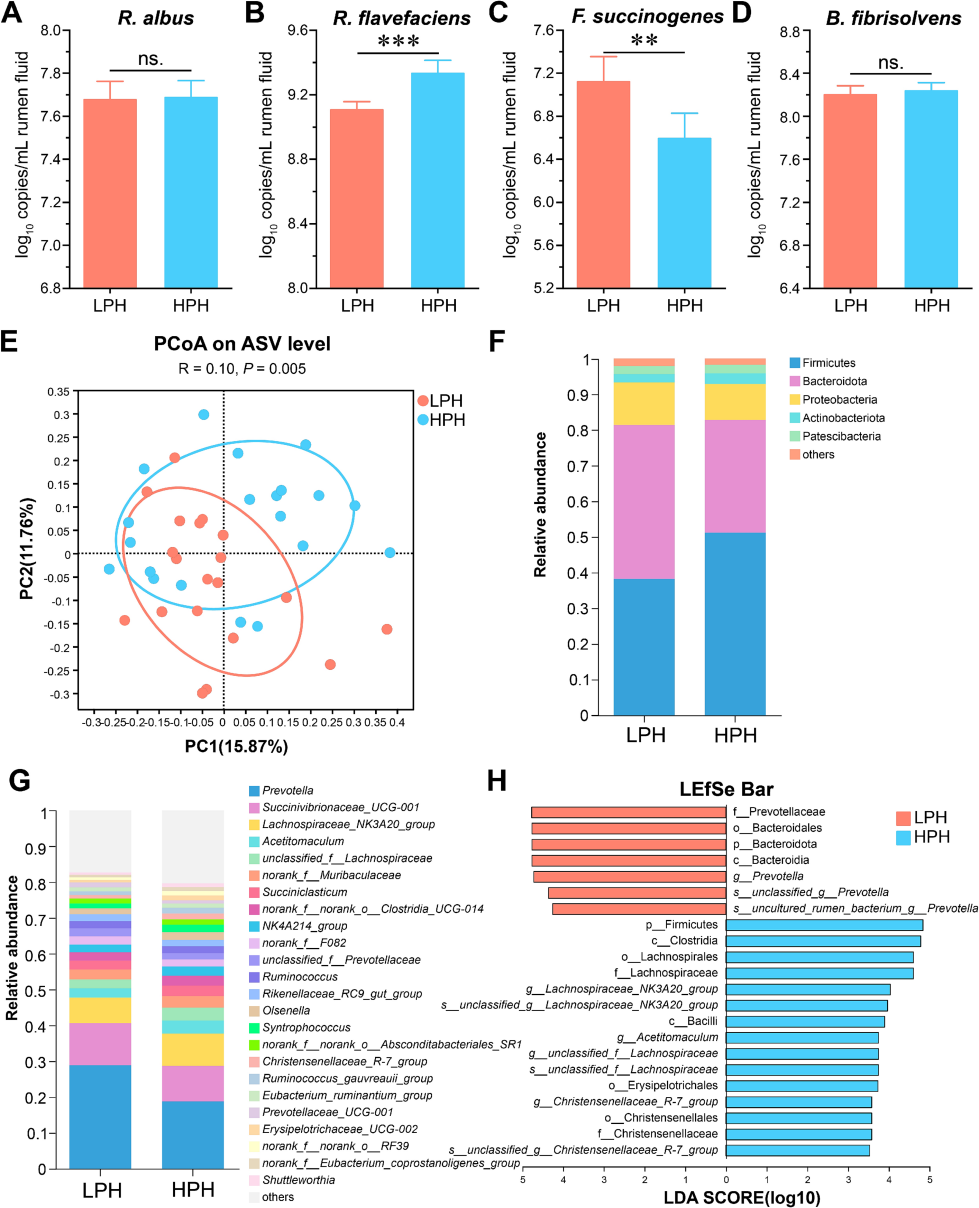
The log_10_ copies per mL rumen fluid of *R. albus* **(A)**, *R. flavefaciens* **(B)**, *F. succinogenes* **(C)**, and *B. fibrisolvens* **(D)** between the LPH and HPH groups. Beta-diversity based on the PCoA **(E)** using the bray curtis distances. Relative abundance of phyla **(F)** and genera **(G)** between two groups. Analysis of differences in the microbial taxa from phylum to species level shown by LEfSe **(H)**. All results are expressed as mean ± SEM. * 0.01 < *p* ≤ 0.05, ** 0.001 < *p* ≤ 0.01, ***p ≤ 0.001.

In order to understand the characteristics of cellulolytic bacteria at different pH, we used qRT-PCR to absolutely quantify some important cellulolytic bacteria reported in previous studies (Li et al., 2017), including *R. albus*, *R. flavefaciens*, *F. succinogenes*, and *B. fibrisolvens* (Figures 1A–D). There were no significant differences in *R. albus* and *B. fibrisolvens* between the LPH and HPH groups. The LPH group decreased the level of *R. flavefaciens* (*p* < 0.001) and increased the level of *F. succinogenes* (*p* = 0.006) compared to the HPH group.

The 16S rRNA gene sequencing showed that 1,741,620 high quality sequences were observed with 43,540 ± 8,549 (mean ± SD) reads per rumen fluid sample. The results revealed the ACE, Chao, Shannon and Simpson index in the LPH group were similar to those in the HPH group (Supplementary Figures S1A–D, Wilcoxon rank sum test). Regarding beta diversity based on the principal coordinate analysis, the LPH group was significantly separated from the HPH group (Figure 1E; ANOSIM, *p* = 0.005). The Venn analysis showed that a total of 20 phyla and 319 genera of bacteria were identified and 18 phyla and 235 genera were shared between the LPH and HPH groups (Supplementary Figures S1E,F). At the phyla and genus level, we further identified the dominant bacteria at each group. Firmicutes (38.24% vs. 51.18%), Bacteroidota (43.15% vs. 31.60%) and Proteobacteria (11.93% vs. 10.08%) were the dominant bacteria in LPH and HPH groups (Figure 1F). At the genus level, the dominant genera among rumen microorganisms of the LPH and HPH groups are *Prevotella* (28.91% vs. 18.81%), *Succinivibrionaceae_UCG-001* (11.74% vs. 9.93%), and *Lachnospiraceae_NK3A20_group* (7.12% vs. 8.99%, Figure 1G).

The results of LEfSe analysis were further applied for differential abundance for phylum level to species between the LPH and HPH groups (Figure 1H, LDA > 3.5, *p* < 0.05). At the phylum level, Bacteroidota and Firmicutes were enriched in the LPH and HPH groups, respectively. At the genus level, the dominant bacteria in the LPH group were *Prevotella*, and the dominant bacteria in the HPH group were *Lachnospiraceae_NK3A20_group*, *Acetitomaculum* and *Christensenellaceae_R-7_group*.

### 3.4 Phenotype and function prediction

**Figure 2:**
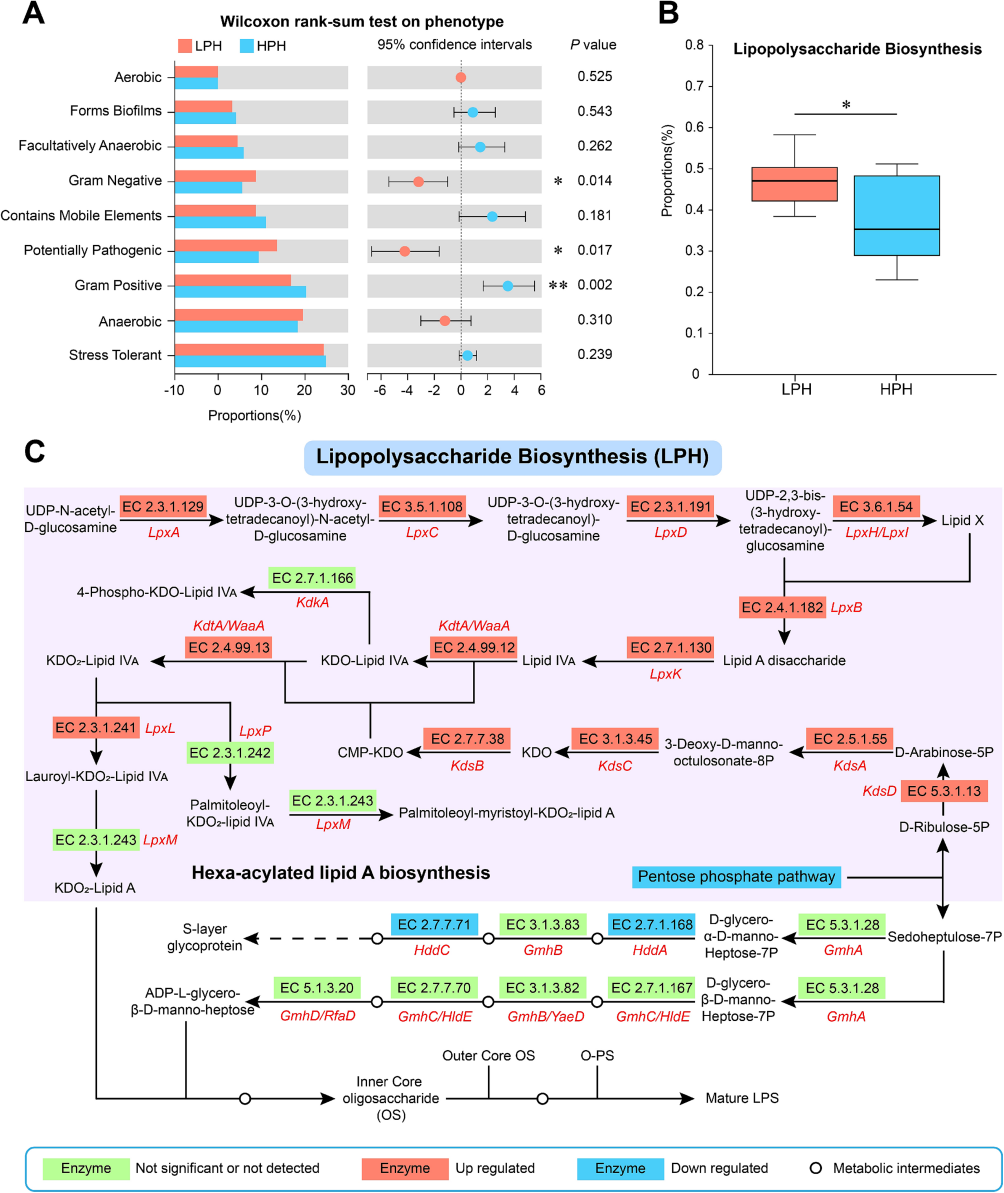
Phenotypic differences between LPH and HPH groups **(A)**. The differences of LPS biosynthetic pathway between LPH and HPH groups **(B)**. Changes in key enzymes of the LPS biosynthetic pathway in the LPH group compared to the HPH group **(C)**. The median is represented by the middle horizontal line in the box plot. The upper and lower lines indicate the maximum and minimum values, respectively, while the upper and lower edges of the box represent the quartiles. * 0.01 < *p* ≤ 0.05, ** 0.001 < *p* ≤ 0.01, ***p ≤ 0.001.

To further understand the effect of pH on LPS biosynthesis, we performed BugBase phenotype prediction to show differences in Gram-negative phenotypes between the two groups. Compared with cows in the HPH group, the proportions of Gram-negative (*p* = 0.014) and potentially pathogenic (*p* = 0.017) phenotypes in the LPH group were significantly increased, while the proportion of Gram-positive (*p* = 0.002) phenotype was significantly reduced (Figure 2A, Wilcoxon rank sum test). Subsequently, we used PICRUSt2 function prediction software at the pathway level 3 and enzyme levels of KEGG (Supplementary Table S1). The results showed that the proportion of LPS biosynthetic pathway was increased in the LPH group relative to the HPH group (Figure 2B, *p* = 0.017). In addition, low pH increased the levels of several enzymes in the LPS biosynthetic pathway, including the *LpxL*. However, the *LpxM* was not significantly affected by pH (Figure 2C).

### 3.5 Correlation analysis of rumen pH, *LpxL*, *LpxM*, serum LPS, and inflammatory markers with rumen microorganisms

**Figure 3:**
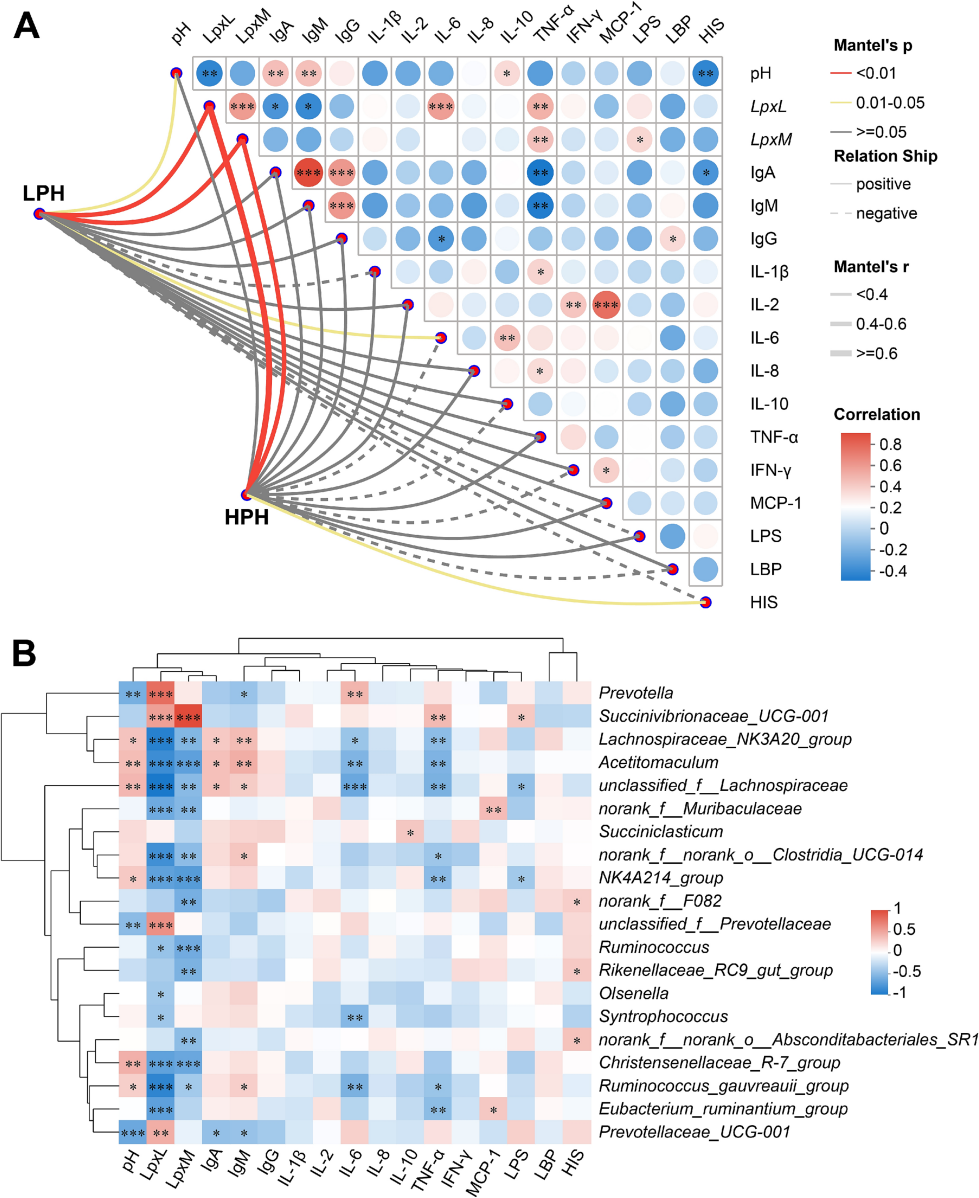
The heatmap on the left depicts the correlation of microbial communities in the LPH and HPH groups with rumen pH, *LpxL*, *LpxM* and serum indicators. The heatmap on the right depicts the correlation between rumen pH, *LpxL*, *LpxM* and serum indicators **(A)**. The heatmap depicts the correlation between rumen pH, *LpxL*, *LpxM* and serum indicators with bacterial genus **(B)**. * 0.01 < *p* ≤ 0.05, ** 0.001 < *p* ≤ 0.01, ***p ≤ 0.001.

In order to present the correlation of microbial communities with pH, *LpxL*, *LpxM*, and serum immune and inflammatory indicators, a Spearman correlation analysis was employed. As shown in Figure 3A, pH was significantly negatively correlated with *LpxL* (*r* = −0.420, *p* = 0.007) and HIS (*r* = −0.419, *p* = 0.007), and significantly positively correlated with IgA (*r* = 0.431, *p* = 0.006), IgM (*r* = 0.424, *p* = 0.006) and IL-10 (*r* = 0.329, *p* = 0.038). *LpxL* showed a significant positive correlation with *LpxM* (*r* = 0.563, *p* < 0.001), IL-6 (*r* = 0.551, *p* < 0.001) and TNF-*α* (*r* = 0.481, *p* = 0.002), but a significant negative correlation with IgA (*r* = −0.342, *p* = 0.031) and IgM (*r* = 0.398, *p* = 0.011). Interestingly, *LpxM*, the key enzyme for hexa-acylated LPS synthesis, also showed a significant positive correlation with serum LPS (*r* = 0.338, *p* = 0.034) and TNF-α (*r* = 0.422, *p* = 0.007). Furthermore, the bacterial communities in both the LPH and HPH groups exhibited a significant positive correlation with *LpxL* and *LpxM* (*r* > 0.432, *p* = 0.001).

As shown in Figure 3B, *Prevotella* was positively correlated with *LpxL* (*r* = 0.814, *p* < 0.001) and IL-6 (*r* = 0.448, *p* = 0.004), but negatively correlated with pH (*r* = −0.490, *p* = 0.001) and IgM (*r* = −0.356, *p* = 0.024). It is noteworthy that *Succinivibrionaceae_UCG-001* demonstrated robust positive correlations with both *LpxL* (*r* = 0.564, *p* < 0.001) and *LpxM* (*r* = 0.998, *p* < 0.001), and exhibited a similar positive correlation with LPS (*r* = 0.346, *p* = 0.029) and TNF-α (*r* = 0.422, *p* = 0.007). Furthermore, a number of potentially beneficial bacteria, such as *Lachnospiraceae_NK3A20_group* and *Acetitomaculum*, exhibited positive correlations with rumen pH, IgA, and IgM (*r* > 0.343, *p* < 0.05), while displaying significant negative correlations with *LpxL*, *LpxM*, IL-6, and TNF-α (*r* < −0.352, *p* < 0.05).

## 4 Discussion

The high intake of high grain diets is one of the most important factors influencing SARA in dairy cows. Previous studies have shown that cows differ in their susceptibility to SARA, even when fed the same diet (Zhang et al., 2022). For this study, cows in the LPH group had high concentrations of TVFA, acetate, propionate, butyrate and valerate. It is widely acknowledged that a correlation exists between the decline in rumen pH during SARA and the accumulation of VFA, with ramifications for lactation performance (Gao and Oba, 2016). However, unlike SARA cows, which experience decreased milk yield, milk fat and milk protein, and increased somatic cell count, the cows in this study did not exhibit these symptoms (Hu et al., 2022; Ma et al., 2022; Meng et al., 2023). Consistently, no significant differences in milk yield, milk fat, milk protein, lactose and total solids between SARA-susceptible and SARA-tolerant cows (Zhang et al., 2022). It can be concluded that the level of SARA risk does not have an additional impact on the lactation performance of the cows in this study. Certainly, we acknowledged that there is a lack of data on individual cow feed intake as a result of the group feeding used in this study. In the future, the inclusion of individual cow feed intake in the study to elucidate its relationship with rumen pH changes will likely better support the findings of this paper.

To investigate the differences in physiological and biochemical functions, immune function, oxidative stress, and inflammatory response between cows in the LPH and HPH groups, we tested serum collected from the tail vein and centrifuged. We found that cows in the LPH group had significantly higher serum levels of BHBA and NEFA compared to those in the HPH group. This suggests that low pH cows are at a higher risk of developing ketosis, negative energy balance and oxidative stress, which is consistent with previous studies (Wu et al., 2023). Additionally, previous studies have shown that SARA increased the concentrations of LPS, HIS and inflammatory factors in ruminants (Chang et al., 2018; Zhao et al., 2018; Wang et al., 2022; Fan et al., 2024). Our findings revealed a notable elevation in LPS, HIS, IL-2, IL-6, and TNF-*α* concentrations at acidic pH levels, accompanied by a discernible tendency toward increased LBP and IL-1β levels. This is consistent with the lower immune status at low pH in this study. The high levels of inflammatory factors in the low pH cows in this study may predict their higher risk of inflammation. Generally, SARA makes cows more susceptible to oxidative stress and hinders milk fat and protein synthesis, and that reactive oxygen species generated during oxidative stress have an activating effect on NLRP3 (Li et al., 2014; Chang et al., 2018; Ma et al., 2022). Our results found that low pH increased the risk of oxidative stress in cows and suppressed mTOR expression. In light of the absence of notable discrepancies in milk fat and protein rates, PI3K, AKT, and NLRP3 at varying rumen pH levels in the present study, further inquiry into the mTOR signaling pathway for milk protein synthesis is warranted. In conclusion, the low pH cows exhibited a greater number of characteristics similar to those observed in cows with SARA with regard to rumen fermentation performance and serum indicators.

During SARA, the population of starch-degrading bacteria increases while the abundance of cellulolytic bacteria decreases in the rumen, such as *R.albus*, *R. flavefaciens*, *F. succinogenes*, and *B. fibrisolvens* (Li et al., 2017). This piqued our interest in studying these cellulolytic bacteria. It is noteworthy that the present study revealed a notable decline in the copy number of *R. flavefaciens* per milliliter of rumen fluid at low pH, accompanied by a notable increase in the copy number of *F. succinogenes*. This may be due to the fact that *F. succinogenes* is Gram-negative and acid-resistant strains may be present. Gram-negative bacteria are vulnerable to lysis and release LPS at prolonged low pH, which is a common characteristic of SARA cows (Monteiro and Faciola, 2020). Research has demonstrated that acid-tolerant *F. succinogenes* S85 strains are capable of digesting corn stover at low pH levels at the same rate as wild-type strains (Wu et al., 2017). The competitive relationship of *F. succinogenes* with *R.albus* and *R. flavefaciens* in the rumen may affect the levels of each cellulolytic bacteria (Yeoman et al., 2021). Therefore, elucidating the relationship between cellulolytic bacteria and pH, as well as the relationship between cellulolytic bacteria in the rumen in the future will help in the research and development of microbial preparations.

Microorganisms are constantly changing throughout a cow’s life cycle and are influencing her health and performance (Zhuang et al., 2024). To further explore the difference of microbial communities in the rumen between the LPH and HPH groups, 16S rRNA gene sequencing and analysis were performed on rumen fluid samples collected from 40 cows. We then analyzed the microbial community characteristics of each group. Our study showed no reduction in bacterial diversity and richness in the LPH group, unlike the SARA cows (Mao et al., 2013; Wu et al., 2023). However, the PCoA plots indicated a significant separation between the LPH and HPH groups, suggesting that the differences in microbial composition between the two groups. At the phylum level, the LPH group had a higher relative abundance of Bacteroidota, while the HPH group had a higher relative abundance of Firmicutes. A growing body of evidence indicates that a reduction in the Firmicutes/Bacteroidota ratio may be linked to inflammatory bowel disease, depression, and breast cancer, and this may be associated with an immune-inflammatory response induced by correlations such as LPS and HIS accumulation (An et al., 2023; Gao et al., 2023; Tai et al., 2024). In this study, the Firmicutes/Bacteroidota ratio was lower in the LPH group compared to the HPH group (0.89 vs. 1.62), which is consistent with higher concentrations of LPS and HIS. At the genus level, consistent with previous studies, *Prevotella* was significantly enriched in the LPH group (Zhang et al., 2022). Given that *Prevotella* is the most prevalent Gram-negative bacterium within the Bacteroidota and its positive correlation with VFA, we were prompted to delve more deeply into the interrelationship between rumen microbes and LPS biosynthesis.

This study employed the BugBase phenotype prediction tool to predict nine phenotypes, including aerobic, forms biofilms, facultatively anaerobic, Gram-negative, contains mobile elements, potentially pathogenic, Gram-positive, anaerobic and stress tolerant. As expected, cows in the LPH group had higher proportions of Gram-negative and potentially pathogenic phenotypes than those in the HPH group, and lower proportions of Gram-positive phenotypes. A decrease in rumen pH not only increases the risk of SARA, but also potentially increases the risk of other underlying diseases (Tufarelli et al., 2024). Given the higher proportion of Gram-negative phenotypes observed in the LPH group and the established association between Gram-negative bacteria and LPS, we elected to concentrate our analysis on the level of LPS synthesis as reflected in the PICRUSt2 prediction results (Douglas et al., 2020). Our results showed significantly higher levels of the LPS synthesis pathway in the LPH group and increased levels of several key enzymes, especially *LpxL*, a key enzyme in the synthesis of penta-acylated LPS. Nevertheless, *LpxM*, a pivotal enzyme engaged in the synthesis of hexa-acylated LPS, did not exhibit notable discrepancies contingent on rumen pH. Therefore, we conducted a correlation analysis between rumen pH, *LpxL*, *LpxM*, serum immune and inflammatory markers with rumen microorganisms. Our results showed that *LpxL* and *LpxM* were positively correlated with inflammatory factors such as IL-6, IL-10 and TNF-*α*, but negatively correlated with IgA and IgM. This suggests that high levels of *LpxL* and *LpxM* may increase the risk of inflammation in dairy cows. Interestingly, we also found that *LpxL* and IL-6 were positively correlated with *Prevotella*, while *LpxL*, *LpxM*, serum LPS, and TNF-α were positively correlated with *Succinivibrionaceae_UCG-001*. It has been demonstrated that the biosynthesis of hexa-acylated LPS necessitates the simultaneous presence of *LpxL* and *LpxM* (Brix et al., 2015). Our results suggested that the source of penta-acylated LPS in the rumen may be related to *Prevotella*, while hexa-acylated LPS may be closely related to *Succinivibrionaceae_UCG-001*. In the future, the structure and function of LPS derived from *Succinivibrionaceae_UCG-001* and its relationship with systemic inflammation in dairy cows still need to be further investigated.

## 5 Conclusion

In conclusion, disorders of acid-base balance of the rumen can alter the rumen fermentation, serum biochemistry, immune and antioxidant functions in dairy cows, and affect rumen microbial composition and LPS biosynthesis. The low pH cows exhibited more characteristics similar to SARA cows, such as increased short-chain fatty acids, elevated levels of inflammatory markers, enhanced oxidative stress and reduced immune function. Low pH induced high abundance of starch-degrading bacteria *Prevotella* and *Succinivibrionaceae_UCG-001* may increase the risk of inflammation in cows by promoting the synthesis and release of penta-acylated LPS and hexa-acylated LPS, respectively, which ultimately enter the peripheral blood circulation and lead to high susceptibility of cows to SARA. The findings of this study enhanced the comprehension of rumen acid-base balance disorders, microbiota, and LPS biosynthesis. This offers a novel perspective on the diagnosis and prevention of SARA and systemic inflammatory responses in dairy cows.

## Data Availability

The data presented in the study are deposited in the NCBI Sequence Read Archive repository, accession number PRJNA1181946.

## References

[ref1] An J. Kwon H. Kim Y. J. (2023). The Firmicutes/Bacteroidetes ratio as a risk factor of breast cancer. J. Clin. Med. 12:2216. doi: 10.3390/jcm12062216, PMID: 36983217 PMC10052522

[ref2] Bevans D. W. Beauchemin K. A. Schwartzkopf-Genswein K. S. McKinnon J. J. McAllister T. A. (2005). Effect of rapid or gradual grain adaptation on subacute acidosis and feed intake by feedlot cattle. J. Anim. Sci. 83, 1116–1132. doi: 10.2527/2005.8351116x, PMID: 15827257

[ref3] Brix S. Eriksen C. Larsen J. M. Bisgaard H. (2015). Metagenomic heterogeneity explains dual immune effects of endotoxins. J. Allergy Clin. Immunol. 135, 277–280. doi: 10.1016/j.jaci.2014.09.036, PMID: 25445821

[ref4] Broderick G. A. Kang J. H. (1980). Automated simultaneous determination of ammonia and total amino acids in ruminal fluid and *in vitro* media. J. Dairy Sci. 63, 64–75. doi: 10.3168/jds.S0022-0302(80)82888-8, PMID: 7372898

[ref5] Chang G. Wang L. Ma N. Zhang W. Zhang H. Dai H.. (2018). Histamine activates inflammatory response and depresses casein synthesis in mammary gland of dairy cows during SARA. BMC Vet. Res. 14:168. doi: 10.1186/s12917-018-1491-3, PMID: 29792195 PMC5966854

[ref6] Chen S. Zhou Y. Chen Y. Gu J. (2018). fastp: an ultra-fast all-in-one FASTQ preprocessor. Bioinformatics 34, i884–i890. doi: 10.1093/bioinformatics/bty560, PMID: 30423086 PMC6129281

[ref7] Douglas G. M. Maffei V. J. Zaneveld J. R. Yurgel S. N. Brown J. R. Taylor C. M.. (2020). PICRUSt2 for prediction of metagenome functions. Nat. Biotechnol. 38, 685–688. doi: 10.1038/s41587-020-0548-6, PMID: 32483366 PMC7365738

[ref8] Dovala D. Rath C. M. Hu Q. Sawyer W. S. Shia S. Elling R. A.. (2016). Structure-guided enzymology of the lipid a acyltransferase LpxM reveals a dual activity mechanism. Proc. Natl. Acad. Sci. USA 113, E6064–E6071. doi: 10.1073/pnas.1610746113, PMID: 27681620 PMC5068295

[ref9] Elmhadi M. E. Ali D. K. Khogali M. K. Wang H. (2022). Subacute ruminal acidosis in dairy herds: microbiological and nutritional causes, consequences, and prevention strategies. Anim. Nutr. 10, 148–155. doi: 10.1016/j.aninu.2021.12.008, PMID: 35702144 PMC9168481

[ref10] Fan S. Zheng M. Ren A. Mao H. Long D. Yang L. (2024). Effects of high-concentrate-induced SARA on antioxidant capacity, immune levels and rumen microbiota and function in goats. Animals (Basel) 14:263. doi: 10.3390/ani14020263 38254432 PMC10812789

[ref11] Fu Y. He Y. Xiang K. Zhao C. He Z. Qiu M.. (2022). The role of rumen microbiota and its metabolites in subacute ruminal acidosis (SARA)-induced inflammatory diseases of ruminants. Microorganisms 10:1495. doi: 10.3390/microorganisms10081495, PMID: 35893553 PMC9332062

[ref12] Gao X. Oba M. (2016). Characteristics of dairy cows with a greater or lower risk of subacute ruminal acidosis: volatile fatty acid absorption, rumen digestion, and expression of genes in rumen epithelial cells. J. Dairy Sci. 99, 8733–8745. doi: 10.3168/jds.2016-11570 27638257

[ref13] Gao M. Wang J. Liu P. Tu H. Zhang R. Zhang Y.. (2023). Gut microbiota composition in depressive disorder: a systematic review, meta-analysis, and meta-regression. Transl. Psychiatry 13:379. doi: 10.1038/s41398-023-02670-5, PMID: 38065935 PMC10709466

[ref14] Han C. Shi C. Liu L. Han J. Yang Q. Wang Y.. (2024). Majorbio cloud 2024: update single-cell and multiomics workflows. iMeta 3:e217. doi: 10.1002/imt2.217, PMID: 39135689 PMC11316920

[ref15] He B. Fan Y. Wang H. (2022). Lactate uptake in the rumen and its contributions to subacute rumen acidosis of goats induced by high-grain diets. Front. Vet. Sci. 9:964027. doi: 10.3389/fvets.2022.964027, PMID: 36204287 PMC9530351

[ref16] Hu X. Li S. Mu R. Guo J. Zhao C. Cao Y.. (2022). The rumen microbiota contributes to the development of mastitis in dairy cows. Microbiol. Spectr. 10:e0251221. doi: 10.1128/spectrum.02512-21, PMID: 35196821 PMC8865570

[ref17] Li M. Penner G. B. Hernandez-Sanabria E. Oba M. Guan L. L. (2009). Effects of sampling location and time, and host animal on assessment of bacterial diversity and fermentation parameters in the bovine rumen. J. Appl. Microbiol. 107, 1924–1934. doi: 10.1111/j.1365-2672.2009.04376.x 19508296

[ref18] Li F. Wang Z. Dong C. Li F. Wang W. Yuan Z.. (2017). Rumen Bacteria communities and performances of fattening lambs with a lower or greater subacute ruminal acidosis risk. Front. Microbiol. 8:2506. doi: 10.3389/fmicb.2017.02506, PMID: 29312208 PMC5733016

[ref19] Li J. Yan F. Chen G. (2014). Reactive oxygen species and NLRP3 inflammasome activation. Ann. Neurol. 75:972. doi: 10.1002/ana.24173 24798299

[ref20] Li S. Yoon I. Scott M. Khafipour E. Plaizier J. C. (2016). Impact of *Saccharomyces cerevisiae* fermentation product and subacute ruminal acidosis on production, inflammation, and fermentation in the rumen and hindgut of dairy cows. Anim. Feed Sci. Technol. 211, 50–60. doi: 10.1016/j.anifeedsci.2015.10.010

[ref21] Liu C. Zhao D. Ma W. Guo Y. Wang A. Wang Q.. (2016). Denitrifying sulfide removal process on high-salinity wastewaters in the presence of Halomonas sp. Appl. Microbiol. Biotechnol. 100, 1421–1426. doi: 10.1007/s00253-015-7039-6, PMID: 26454867

[ref22] Ma N. Abaker J. A. Wei G. Chen H. Shen X. Chang G. (2022). A high-concentrate diet induces an inflammatory response and oxidative stress and depresses milk fat synthesis in the mammary gland of dairy cows. J. Dairy Sci. 105, 5493–5505. doi: 10.3168/jds.2021-21066 35346479

[ref23] Magoč T. Salzberg S. L. (2011). FLASH: fast length adjustment of short reads to improve genome assemblies. Bioinformatics 27, 2957–2963. doi: 10.1093/bioinformatics/btr507, PMID: 21903629 PMC3198573

[ref24] Mao S. Y. Zhang R. Y. Wang D. S. Zhu W. Y. (2013). Impact of subacute ruminal acidosis (SARA) adaptation on rumen microbiota in dairy cattle using pyrosequencing. Anaerobe 24, 12–19. doi: 10.1016/j.anaerobe.2013.08.003, PMID: 23994204

[ref25] Meng M. Li X. Huo R. Ma N. Chang G. Shen X. (2023). A high-concentrate diet induces mitochondrial dysfunction by activating the MAPK signaling pathway in the mammary gland of dairy cows. J. Dairy Sci. 106, 5775–5787. doi: 10.3168/jds.2022-22907 37296051

[ref26] Monteiro H. F. Faciola A. P. (2020). Ruminal acidosis, bacterial changes, and lipopolysaccharides. J. Anim. Sci. 98:skaa248. doi: 10.1093/jas/skaa248, PMID: 32761212 PMC7455920

[ref27] Plaizier J. C. Li S. Tun H. M. Khafipour E. (2016). Nutritional models of experimentally-induced subacute ruminal acidosis (SARA) differ in their impact on rumen and hindgut bacterial communities in dairy cows. Front. Microbiol. 7:2128. doi: 10.3389/fmicb.2016.02128, PMID: 28179895 PMC5265141

[ref28] Plaizier J. C. Mulligan F. J. Neville E. W. Guan L. L. Steele M. A. Penner G. B. (2022). Invited review: effect of subacute ruminal acidosis on gut health of dairy cows. J. Dairy Sci. 105, 7141–7160. doi: 10.3168/jds.2022-21960, PMID: 35879171

[ref29] Rivera-Chacon R. Castillo-Lopez E. Ricci S. Petri R. M. Reisinger N. Zebeli Q. (2022). Supplementing a phytogenic feed additive modulates the risk of subacute rumen acidosis, rumen fermentation and systemic inflammation in cattle fed acidogenic diets. Animals 12:1201. doi: 10.3390/ani12091201, PMID: 35565627 PMC9105827

[ref30] Soest P. J. Robertson J. B. Lewis B. A. (1991). Methods for dietary fiber, neutral detergent fiber, and nonstarch polysaccharides in relation to animal nutrition. J. Dairy Sci. 74, 3583–3597. doi: 10.3168/jds.s0022-0302(91)78551-2, PMID: 1660498

[ref31] Tai W. C. Yao C. C. Tsai Y.-C. (2024). P1219 changes in gut microbiota of patients with inflammatory bowel disease receiving biologic therapy. J. Crohn's Colitis 18:i2166. doi: 10.1093/ecco-jcc/jjad212.1349

[ref32] Tufarelli V. Puvača N. Glamočić D. Pugliese G. Colonna M. A. (2024). The Most important metabolic diseases in dairy cattle during the transition period. Animals (Basel) 14:816. doi: 10.3390/ani14050816, PMID: 38473200 PMC10930595

[ref33] Wang K. Sun Z. Li Y. Liu M. Loor J. J. Jiang Q.. (2022). Histamine promotes adhesion of neutrophils by inhibition of autophagy in dairy cows with subacute ruminal acidosis. J. Dairy Sci. 105, 7600–7614. doi: 10.3168/jds.2022-22036, PMID: 35940921

[ref34] Wu Z. Guo Y. Zhang J. Deng M. Xian Z. Xiong H.. (2023). High-dose vitamin E supplementation can alleviate the negative effect of subacute ruminal acidosis in dairy cows. Animals (Basel) 13:486. doi: 10.3390/ani13030486, PMID: 36766375 PMC9913405

[ref35] Wu C.-W. Spike T. Klingeman D. M. Rodriguez M. Bremer V. R. Brown S. D. (2017). Generation and characterization of acid tolerant *Fibrobacter succinogenes* S85. Sci. Rep. 7:2277. doi: 10.1038/s41598-017-02628-w, PMID: 28536480 PMC5442110

[ref36] Yeoman C. J. Fields C. J. Lepercq P. Ruiz P. Forano E. White B. A.. (2021). *In vivo* competitions between *Fibrobacter succinogenes*, Ruminococcus flavefaciens, and Ruminoccus albus in a Gnotobiotic sheep model revealed by multi-Omic analyses. MBio 12:e03533-20. doi: 10.1128/mbio.03533-20 33658330 PMC8092306

[ref37] Zebeli Q. Dijkstra J. Tafaj M. Steingass H. Ametaj B. N. Drochner W. (2008). Modeling the adequacy of dietary fiber in dairy cows based on the responses of ruminal pH and milk fat production to composition of the diet. J. Dairy Sci. 91, 2046–2066. doi: 10.3168/jds.2007-0572, PMID: 18420634

[ref38] Zeng J. Lv J. Duan H. Yang S. Wu J. Yan Z.. (2023). Subacute ruminal acidosis as a potential factor that induces endometrium injury in sheep. Int. J. Mol. Sci. 24:1192. doi: 10.3390/ijms24021192, PMID: 36674716 PMC9861559

[ref39] Zhang Z. A. Li F. Ma Z. Y. Li F. D. Wang Z. L. Li S. R.. (2023). Variability in chewing, ruminal fermentation, digestibility and bacterial communities between subacute ruminal acidosis-susceptible and acidosis-tolerant sheep. Animal 17:100902. doi: 10.1016/j.animal.2023.100902 37544054

[ref40] Zhang T. Mu Y. Zhang R. Xue Y. Guo C. Qi W.. (2022). Responsive changes of rumen microbiome and metabolome in dairy cows with different susceptibility to subacute ruminal acidosis. Anim. Nutr. 8, 331–340. doi: 10.1016/j.aninu.2021.10.009, PMID: 35024470 PMC8718735

[ref41] Zhang H. Xue Y. Xie W. Wang Y. Ma N. Chang G.. (2023). Subacute ruminal acidosis downregulates FOXA2, changes oxidative status, and induces autophagy in the livers of dairy cows fed a high-concentrate diet. J. Dairy Sci. 106, 2007–2018. doi: 10.3168/jds.2022-22222, PMID: 36631320

[ref42] Zhao C. Liu G. Li X. Guan Y. Wang Y. Yuan X.. (2018). Inflammatory mechanism of Rumenitis in dairy cows with subacute ruminal acidosis. BMC Vet. Res. 14:135. doi: 10.1186/s12917-018-1463-7, PMID: 29673406 PMC5909223

[ref43] Zhao W. Shen T. Zhao B. Li M. Deng Z. Huo Y.. (2024). Epigallocatechin-3-gallate protects bovine ruminal epithelial cells against lipopolysaccharide-induced inflammatory damage by activating autophagy. J. Anim. Sci. Biotechnol. 15:109. doi: 10.1186/s40104-024-01066-9, PMID: 39118120 PMC11311925

[ref44] Zhuang Y. Liu S. Gao D. Xu Y. Jiang W. Chen T.. (2024). The Bifidobacterium-dominated fecal microbiome in dairy calves shapes the characteristic growth phenotype of host. NPJ Biofilms Microb. 10:59. doi: 10.1038/s41522-024-00534-4 10.1038/s41522-024-00534-4 PMC11271470 39034349

