# Supplementary figures and images for: Disorders of acid-base balance promote rumen lipopolysaccharide biosynthesis in dairy cows by modulating the microbiome
Source: Front Microbiol. 2024 Nov 14;15:1492476. doi: 10.3389/fmicb.2024.1492476 (PMC11604126; doi:10.3389/fmicb.2024.1492476)

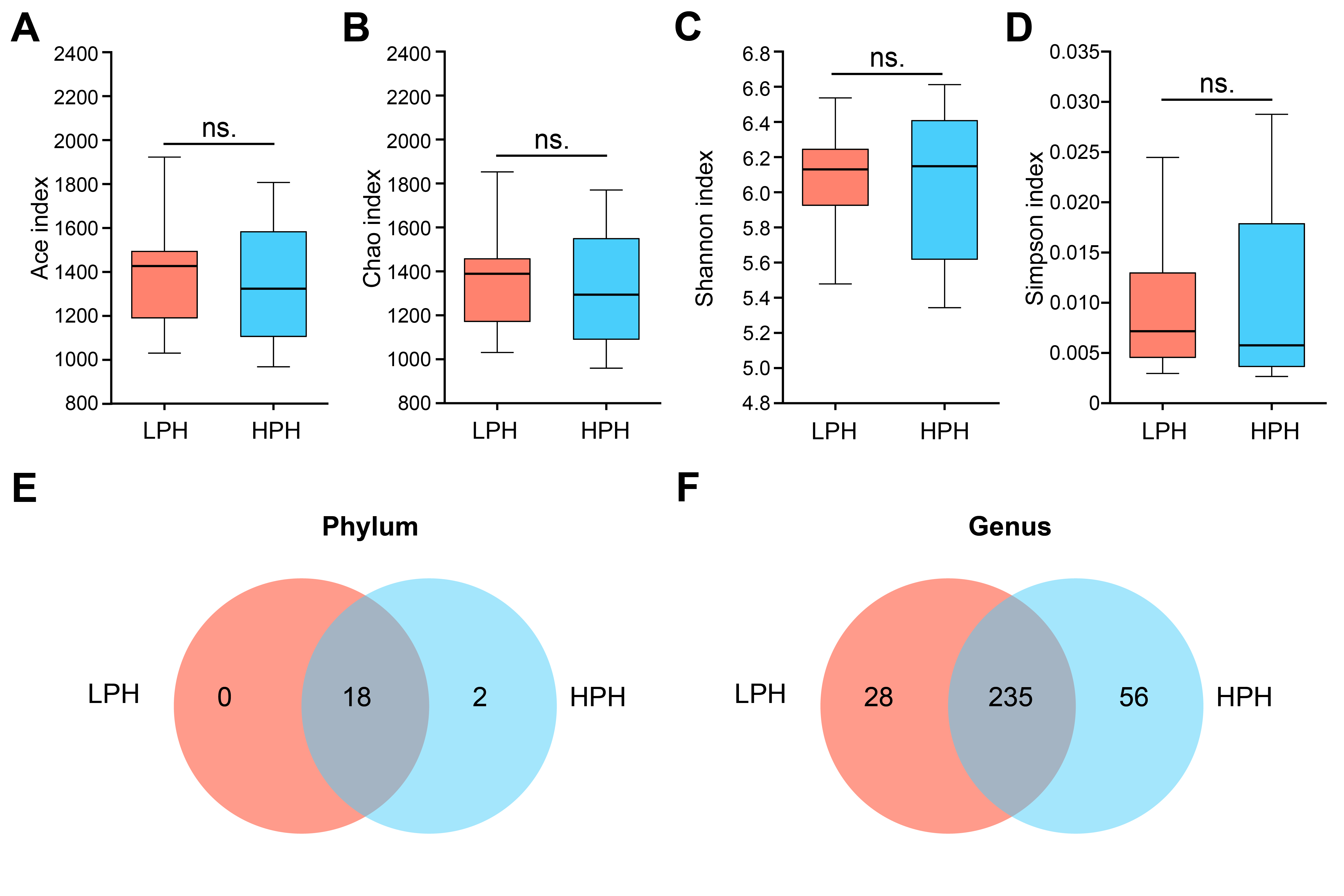

Supplement: Supplementary file 2 [file Image_1.JPEG]
